# Regarding the vitamin D and thyroid function of pregnant women in a sunny region, is there any connection?

**DOI:** 10.1590/1806-9282.20250694

**Published:** 2026-03-30

**Authors:** Ilker Sengul, Demet Sengul

**Affiliations:** 1 Giresun University, Faculty of Medicine, Division of Endocrine Surgery - Giresun, Turkey.; 2 Giresun University, Faculty of Medicine, Department of General Surgery - Giresun, Turkey.; 3 Giresun University, Faculty of Medicine, Department of Pathology - Giresun, Turkey.

Dear Editor,

Thyroidology is a vital and dynamic discipline that deals not just with the disorders and therapeutic options of the delicate papillon gland but also with Gynecologic Endocrinology, which requires a graceful approach^1,2,3,4,5,6,7,8,9,10,11,12^. We read a great deal of the article by Almeida et al.^13^ entitled “Vitamin D and thyroid function of pregnant women in a sunny region: is there any connection?” published in volume 71, Rev Assoc Med Bras. The study purposed to investigate the association between serum vitamin D concentrations and thyroid hormone levels in pregnant women residing in a sunny region of Northeast Brazil. The central finding of no significant correlation or association between vitamin D levels and thyroid hormones is a notable contribution to the ongoing discussion on this topic. The study’s focus on a pregnant population in a sunny region is a particular strength, offering a unique perspective compared to studies conducted in areas with less sunlight exposure. Furthermore, the finding of a high prevalence of vitamin D insufficiency despite the sunny location raises important questions about the factors influencing vitamin D status during pregnancy, even in such environments. The authors appropriately highlighted the potential influence of region-specific and environmental factors on the relationship between vitamin D and thyroid function. However, while the study provides valuable data, several aspects warrant further critical consideration. Firstly, the cross-sectional design of the study inherently might limit the ability to establish causal relationships between vitamin D status and thyroid hormone levels. Therefore, while the study found no association, it cannot definitively rule out the possibility of a longitudinal or dynamic relationship between these factors during pregnancy, as suggested by a study in China,which observed associations that varied across gestational trimesters. Secondly, the single-center setting and the convenient sampling method may limit the generalizability of the findings to broader populations of pregnant women, even within Northeast Brazil. Including participants from multiple centers with varying sociodemographic and lifestyle factors could provide a more comprehensive understanding. Thirdly, the exclusion of women with known thyroid disorders or those using multivitamin supplements or medications containing iodine might have introduced a selection bias. While this exclusion aimed to minimize confounding factors, it also means the study’s findings may not be fully representative of the entire pregnant population, including those more susceptible to thyroid dysfunction or vitamin deficiencies. Moreover, while this valued study collected data on several covariates, the self-reported nature of salt intake might introduce recall bias and may not accurately reflect iodine status, even though only those with adequate iodine status were included in the subanalysis. More objective measures of iodine intake could strengthen future investigations. The study also acknowledges that unmeasured factors such as sunscreen use and dietary intake might have influenced the results. Given the sunny location, sunscreen use could be a significant factor affecting vitamin D synthesis, and detailed dietary assessments could provide insights into vitamin D intake from sources other than sunlight. Future research should consider incorporating these variables. Finally, the ongoing debate regarding the optimal definition of vitamin D sufficiency and insufficiency should be remarked on. The authors had utilized commonly accepted cut-offs, but the high rate of insufficiency regarding these criteria, even in a sunny region, underscores the need for continued discussion and research to determine the most appropriate vitamin D targets during pregnancy for optimal maternal and fetal health. In conclusion, the study by Almeida et al provides valuable region-specific data on vitamin D and thyroid function in pregnant women. However, the limitations of its cross-sectional design, single-center setting, and exclusion criteria might warrant a cautious interpretation of the findings. Future research employing longitudinal designs, multi-center recruitment, and the inclusion of factors such as sunscreen use and detailed dietary data would be beneficial to further elucidate the complex relationship between vitamin D status and thyroid health during pregnancy across diverse populations and regions. This issue merits further investigation. We thank Almeida et al.^13^ for their valuable study on vitamin D and pregnancy in Thyroidology.

## Data Availability

The datasets generated and/or analyzed during the current study are available from the corresponding author upon reasonable request.
